# Regional education and wealth-related inequalities in malnutrition among women in Bangladesh

**DOI:** 10.1017/S1368980021003840

**Published:** 2022-06

**Authors:** Sorif Hossain, Md Mohsan Khudri, Rajon Banik

**Affiliations:** 1 Institute of Statistical Research and Training, University of Dhaka, Dhaka, Bangladesh; 2 Department of Economics, Fogelman College of Business and Economics, The University of Memphis, 3675 Central Ave, Memphis, TN 38152, USA; 3 Department of Public Health and Informatics, Jahangirnagar University, Savar, Dhaka, Bangladesh

**Keywords:** BMI, Underweight, Overweight, Obesity, Socioeconomic inequalities, Regional variation, Bangladesh

## Abstract

**Objectives:**

This paper examines the associations of socio-economic and demographic correlates with malnutrition among women and investigates education and wealth-related inequalities in malnutrition among women by region.

**Design:**

We utilise a two-level mixed-effects logistic regression model to evaluate the associations and employ the concentration, Wagstaff and Erreygers’s correction indices to measure socio-economic inequalities in malnutrition among women.

**Setting:**

Bangladesh Demographic and Health Survey data.

**Participants:**

Non-pregnant women aged 15–49 years.

**Results:**

We find evidence of a significant cluster effect in the data. Women’s age, marital status, total children ever born, education level, husband’s/partner’s education level, residence and wealth index appear to be significantly associated with women underweight and overweight/obesity status. Underweight status is higher among less-educated women and women from poor households, whereas overweight/obesity is more concentrated among higher educated women and women from wealthy households. The southwestern region of the country demonstrates lower education and wealth-related inequalities in malnutrition among women. In contrast, the central and the northeastern areas apparently experience the highest education and wealth-related inequalities in malnutrition among women. The regional differences in predicted probabilities of being underweight shrink at higher education level and the richest quintile, whereas the differences in overweight/obese diminish at the primary education level and lower quintile households.

**Conclusions:**

Our findings strengthen the evidence base for effective regional policy interventions to mitigate education and wealth-related inequalities in malnutrition among women. There is a need for developing regional awareness programmes and establishing regional monitoring cells to ensure proper health and nutrition facilities in underprivileged regions.

The spectrum of malnutrition includes both undernutrition (BMI or BMI ≤ 18·5 kg/m^2^) and overweight (25 ≤ BMI < 30 kg/m^2^) or obesity (BMI ≥ 30 kg/m^2^)^(1)^. Underweight and overweight/obesity have traditionally been regarded as distinct public health concerns, but rising international research shows that underweight and overweight/obesity may coexist in smaller demographics^(2,3)^. Almost one-third of the population worldwide suffers from at least one type of malnutrition (underweight, overweight/obesity)^(4,5)^. Globally in 2016, more than 600 million adults are underweight^(6)^, while 1·9 billion (or 39 % of the total population) are overweight/obese^(7)^. The WHO defines the coexistence of undernutrition with overweight and obesity within the same population as the ‘double burden of malnutrition’^(5)^. Like many other South-East Asian emerging countries, Bangladesh has begun to suffer a double burden of persistently high rates of undernutrition and rising rates of overweight and obesity^(8,9)^.

Bangladesh has been demonstrating significant improvement in malnutrition among women since the last couple of decades; nevertheless, progress might not be the same, depending on various factors^(10)^. So priority must be given to identifying risks for individuals and households influenced by demographic and socio-economic status (SES hereafter)^(11)^. Age is positively associated with overweight and obesity relative to normal weight, and the younger women are more likely to be underweight compared with the elder women^(12,13)^. Women who are currently married are much less overweight than those who are not^(13)^. Similarly, the non-married (e.g. divorced, widowed or separated) women are less likely to be overweight than married women^(9,13)^. Women who give birth to their first child before 18 are less likely to be underweight^(13)^. The women with fewer children aged under 5 years in the household are less likely to be underweight and more likely to be overweight than those who have more^(13)^. A study conducted on Bangladeshi women finds that exposure to underweight is higher among blue-collar workers, e.g. garment workers and women whose partners are involved in blue-collar job partners^(14)^. Another study reports that women with female household heads are more likely to be overweight as compared with women with male household heads^(15)^. Past research finds that watching television and contraceptive use are associated with overweight and obesity in urban and rural areas^(15)^. Islam *et al.* (2020) find that obesity is associated with a high socio-economic position, a low educational level, inadequate physical activity and middle age^(16)^. On the other hand, women with young age, single women (i.e. widowed or divorced or separated) and being a member of a larger family are associated with an increased risk of being underweight^(17)^.

There are significant variations in the prevalence of several health indicators attributable to SES differences in urban and rural areas of Bangladesh^(18,19)^. Rural women are less likely to be overweight and obese while more likely to be underweight relative to urban women, respectively^(12)^. Women’s educational attainment is significantly negatively associated with being underweight and positively associated with pre-overweight and overweight compared with normal weight^(20)^. A couple of studies show that women from the richest quintile are more prone to being overweight and obese than the poorest women^(8,21)^. Kamal *et al.* (2015) demonstrate that the risk of being underweight increases gradually with the decreases in the wealth index, while the risk of being overweight decreases significantly with the rise in the standard of living index^(20)^. Meanwhile, the burden of chronic energy deficiency among women from low-income families throughout this country has been very high (rural 38·8% *v*. urban 29·7 %)^(22)^.

Regional and socio-economic differences in undernutrition and overnutrition have emerged as a matter of concern, considering growing evidence of the strong persistence of inequalities^(9,23,24)^. Urban people are in better condition concerning many socio-demographic aspects compared with their rural counterparts^(25)^. Investigating the regional variation in malnutrition among women can provide a new lens for policymakers to develop regional inequality strategies in the SES. Studies from lower and middle-income countries like Bangladesh^(23)^, Pakistan^(24)^ and Nepal^(26)^ also find substantial geographical differences in the prevalence of malnutrition. In Bangladesh, malnutrition rates are higher in the northeastern region than in the country’s northwestern part, despite lower poverty rates^(19,27)^. Similarly, although having lower income poverty, the eastern region has distinct non-income features. For example, the Chittagong region has various hill trails and coastal belts, whereas the Sylhet region contains ecologically vulnerable areas marked by physical isolation, wetland habitats and social conservatism^(19,23)^. In rural and urban settings, women from households with poor economic status are less likely to be overweight with respect to those from affluent households^(28)^.

There has been a dearth of research to assess the regional inequalities in malnutrition. Only a few notable studies examine regional patterns in the association between SES and health outcomes in Bangladesh^(29,30)^. No studies assess the regional inequalities in malnutrition among women. Also, to the best of our knowledge, no study has yet analysed the regional variation in education and wealth-related inequalities, particularly in underweight, overweight and obesity among reproductive women in Bangladesh. This paper has two primary objectives. First, we examine the associations of socio-economic and demographic correlates with underweight and overweight/obesity of ever-married women aged 15–49 years in Bangladesh. The significant contribution of this paper is to investigate if the association between women’s education level, household wealth index and extreme categories of BMI, i.e. underweight, overweight and obesity, differs by region. Second, we determine the degree of wealth and education-related inequalities in underweight, overweight and obesity among women across regions. We endeavour to contribute to the existing literature by examining this interesting issue.

## Methods

### Data and sample design

The current study uses repeated cross-sectional data of the most recent four rounds (2007, 2011, 2014 and 2017–2018) of the Bangladesh Demographic and Health Survey (BDHS) from the DHS website^(31)^ and pooled the data sets.

All four surveys adopt a two-stage stratified sampling technique to select households. The last three surveys use the sampling frame of the 2011 Population and Housing Census of the People’s Republic of Bangladesh^(18)^, while the 2007 BDHS uses the frame from the 2001 Population Census. The sampling process involves stratifying the sample into regions and breaking down parts further into urban and rural areas. The enumeration areas (EA), also known as clusters, for each survey are selected in the first stage employing the probability proportional to size sampling technique. The BDHS conducted in 2007, 2011, 2014 and 2017–18 include 675, 600, 600 and 361 EAs. A household listing prepared in all selected clusters is utilised as a sampling frame to select households in the second stage. Using an equal probability systematic sampling technique, 30 households, on average, are drawn from each selected cluster. After purging pregnant women and women with missing information on the key outcome variable, i.e. BMI and other covariates from each survey data set, we retain 55, 248 non-pregnant women aged 15–49 years in the final analysis.

### Outcome variable

Women underweight and overweight/obesity are the outcome variables in the current study. Due to insufficient survey data for overweight and obesity, we combine them as one category and named overweight/obesity. We calculate underweight and overweight/obesity from women’s BMI. We define women with BMI < 18 50 kg/m^2^ as underweight and women with BMI 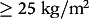 as overweight/obese^(19)^.

### Predictor variables

The independent variables of the current study include age groups of women (in years), sex of household head, current marital status, the total number of children ever born, age of the respondent at first birth (in years), women’s highest education level, husband’s/partner’s highest education level, women’s occupation, husband’s/partner’s occupation, region, place of residence and wealth index. The BDHS segment women’s age into seven groups: 15–19, 20–24, 25–29, 30–34, 35–39, 40–44, and 45–49. We define women as currently single if they are divorced, separated or widowed during interview. The total number of children ever born is segmented into three categories: 0–2, 3–5 and 5+. Next, we categorise the respondent’s age at first birth into four groups: 10–15, 16–20, 21–25, 26–30 and 30+. The BDHS represents the highest education level of women as well as their husband/partner with four categories: No education, Primary, Secondary and Higher. No education indicates that the respondent never attended school, primary education ranges from grades 1 to 5, secondary education refers to grades 6 to 10 and higher education implies grades 11 and above. We categorise the occupation of women as well as their husband/partner into three groups: unemployed, white-collar job and blue-collar job. We consider all types of desk jobs as white-collar jobs (professional/technical/managerial/business) and other works requiring physical labour as blue-collar jobs (agriculture/household and domestic/sales/services/skilled and unskilled manual). Retired or student or unemployed individuals are classified as unemployed. Bangladesh comprises eight administrative regions, known as division, such as Barisal, Chittagong, Dhaka, Khulna, Mymensingh, Rajshahi, Rangpur and Sylhet. Note that the 2011 and the 2017–2018 BDHS include Rangpur and Mymensingh, respectively, as a new division for the first time in the BDHS data sets. Moreover, the survey classifies the type of residence into two groups: rural and urban areas. The wealth index segments households into five groups based on its selected assets and characteristics: poorest (reference category), poorer, middle, richer and richest. The wealth index is constructed using principal component analysis (PCA).

### Statistical analysis

We run the *χ* ^2^ test of independence and ANOVA to examine if demographic and socio-economic factors have a significant statistical association with outcome variables, i.e. underweight and overweight/obese. Next, we employ a two-level mixed-effects logistic regression model to identify the socio-economic and demographic correlates of underweight and overweight/obesity and evaluate their association with the outcome variables. Our adjusted models include the interaction terms between region and education and, also, between region and wealth to examine if the association between women’s education level, household wealth index and malnutrition among women indicators differ by region. The following models are estimated.

Our empirical model for underweight is below.

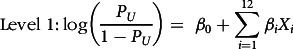




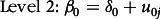




Our empirical model for overweight/obesity is below.

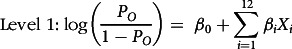




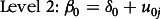




Here, P_U_ and P_O_ are the probability of being underweight and overweight/obese, respectively, and 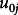 is the random cluster effect. We evaluate multicollinearity by calculating tolerance and variance inflation factor. A tolerance of 0·1 or less and a variance inflation factor of 10 or greater is a cause for concern. We compute the intraclass correlation coefficient to determine the suitability of the multilevel model. The intraclass correlation coefficient ranges from 0 to 1. The model with an intraclass correlation coefficient value greater than 0 indicates that the multilevel analysis is appropriate for the analysis. We report several goodness-of-fit indicators, including the log-likelihood, Wald- 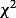 and likelihood ratio test (Table 1).

**Table 1:** Multilevel logistic regression analysis on socio-economic and demographic correlates of extreme categories of BMI among 15–49 aged women

Correlates	Underweight		Overweight/Obesity	
	OR	95 % CI	OR	95 % CI
Age (Ref: 15–19)				
20–24	0·59***	0·54, 0·65	2·19***	1·89, 2·53
25–29	0·35***	0·32, 0·39	4·23***	3·66, 4·88
30–34	0·25***	0·23, 0·28	6·44***	5·56, 7·46
35–39	0·22***	0·20, 0·25	7·80***	6·71, 9·07
40–44	0·23***	0·21, 0·27	7·89***	6·76, 9·21
45–49	0·25***	0·22, 0·28	8·13***	6·93, 9·53
Sex of household head (Ref: Male)				
Female			1·04	0·97, 1·11
Current marital status (Ref: Single)				
Married	0·55***	0·50, 0·61	1·59***	1·41, 1·79
Total children ever born (Ref: 0–2)				
3–5	1·20***	1·12, 1·28	0·84***	0·78, 0·90
5+	1·67***	1·50, 1·86	0·58***	0·52, 0·65
Age at first birth (Ref: 10–15)				
16–20	1·09**	1·03, 1·16	0·88***	0·82, 0·93
21–25	1·26***	1·16, 1·38	0·80***	0·74, 0·87
26–30	1·41***	1·17, 1·69	0·77*	0·67, 0·89
30+	1·35	0·92, 1·99	0·70**	0·53, 0·92
Education level (Ref: No education)				
Primary	0·78***	0·73, 0·84	1·32***	1·23, 1·41
Secondary	0·63***	0·58, 0·69	1·54***	1·42, 1·67
Higher	0·48***	0·41, 0·57	1·66***	1·48, 1·86
Husband’s/Partner’s education (Ref: No education)				
Primary	0·90***	0·84, 0·95	1·21***	1·07, 1·23
Secondary	0·82***	0·77, 0·89	1·26***	1·16, 1·34
Higher	0·72***	0·64, 0·82	1·41***	1·32, 1·59
Occupation (Ref: Unemployed)				
White-collar job	0·89	0·77, 1·02	0·92	0·83, 1·03
Blue-collar job	1·41***	1·33, 1·50	0·61***	0·58, 0·64
Husband’s/Partner’s occupation (Ref: Unemployed)				
White-collar job	0·92	0·69, 1·22	0·97	0·79, 1·21
Blue-collar job	1·15	0·87, 1·52	0·76**	0·61, 0·95
Region (Ref: Barisal)				
Chittagong	0·91	0·81, 1·01	1·09*	0·98, 1·21
Dhaka	1·05	0·95, 1·18	0·94	0·85, 1·04
Khulna	0·81***	0·72, 0·91	1·07	0·96, 1·19
Mymensingh	0·84**	0·72, 0·99	1·08	0·93, 1·25
Rajshahi	0·98	0·88, 1·09	0·99	0·89, 1·09
Rangpur	0·90*	0·80, 1·01	0·90*	0·81, 1·01
Sylhet	1·71***	1·53, 1·91	0·66***	0·59, 0·74
Place of residence (Ref: Urban)				
Rural	1·13***	1·07, 1·21	0·77***	0·73, 0·81
Wealth index (Ref: Poorest)				
Poorer	0·78***	0·73, 0·84	1·24***	1·13, 1·36
Middle	0·66***	0·61, 0·71	1·61***	1·48, 1·76
Richer	0·53***	0·49, 0·57	2·18***	1·99, 2·32
Richest	0·27***	0·25, 0·30	4·02***	3·65, 4·42
Random effect variance	0·04***	0·27, 0·60	0·03***	0·022, 0·050
ICC	0·011	0·081, 0·018	0·010	0·006, 0·015
Log-likelihood	−21 550·833		−23 998·784	
Wald - 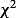 †	<0·001		<0·001	
Likelihood ratio test†	<0·001		<0·001	

The significance level of OR:

* *P* < 0·1.

† *P*-value of the test.

** *P* < 0·05.

*** *P* < 0·01.

We employ the Concentration index (CI), Wagstaff’s Index (WI) and Erreygers’s correction (EI) index to measure socio-economic inequalities in the underweight and overweight/obesity status of women. The CI ranges from −1 to +1. It provides a summary statistic in measuring socio-economic inequalities in the health sector^(32,33)^. The index’s negative values indicate that the variable of interest is higher, on average, among the poor, while positive values indicate concentration among the better off. A zero value of the CI represents no socio-economic inequality in women underweight and overweight/obesity status, respectively. The larger in absolute size the index is, the greater the degree of inequality^(34)^. We assess the regional variation in outcome variables through Wagstaff’s normalization index (WI) and Erreygers’s correction (Erreygers Index or EI)^(34,35)^. We use CI to examine the regional variation through the geographical map. All three indices are defined as follows^(34,35)^:

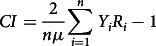




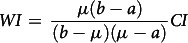




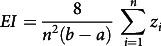

where 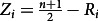 .

For µ > 0, when the poorest (non-educated) *i* individuals have a value of Y equal to zero and the richest (higher educated) *n-i* individuals have a value of Y equal to 1, then the CI is maximum. Here n is the required sample size, b is the maximum, a is the minimum rank values and R is the individual’s fractional rank in the socio-economic status distribution.

We adjust all the estimates using the STATA command ‘svy', including two-stage sampling weight, cluster and strata provided by BDHS. We perform statistical analyses using STATA 14.0^(36)^ and R (version 4.0.0).

## Results

Of the 55 248 women included in the current study, about 19 % and 25 % are underweight and overweight/obese, respectively. Table 2 reports the prevalence and bivariate analysis of women’s underweight and overweight/obesity status by socio-economic and demographic predictors. Woman’s age, current marital status, the total number of children ever born, age at first birth, education level, husband’s/partner’s education level, occupation, husband’s/partner’s occupation, region, place of residence and wealth index show significant association with both underweight and overweight/obesity status of women. However, the sex of the household head exhibits a significant association only with women’s overweight/obese status.

**Table 2:** Bivariate analysis on women’s underweight and overweight/obesity by socio-economic and demographic correlates

Demographic characteristics	Underweight		Overweight/obesity	
	Yes	No	Yes	No
Age (in years)				
15–19	0·30	0·70	0·08	0·92
20–24	0·23	0·77	0·16	0·84
25–29	0·17	0·83	0·24	0·76
30–34	0·15	0·85	0·30	0·70
35–39	0·15	0·85	0·30	0·70
40–44	0·18	0·82	0·29	0·71
45–49	0·19	0·81	0·24	0·76
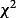	762·64		1608·34	
*P*-value	<0·001		<0·001	
Sex of household head				
Male	0·19	0. 81	0·23	0·77
Female	0·19	0. 81	0·27	0·73
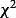	0·063		27·90	
*P*-value	0·844		<0·001	
Current marital status				
Single	0·27	0·73	0·20	0·80
Married	0·19	0·81	0·24	0·76
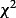	157·20		36·63	
*P*-value	<0·001		<0·001	
Total children ever born				
0–2	0·19	0·81	0·24	0·76
3–5	0·18	0·82	0·25	0·75
5+	0·27	0·73	0·15	0·85
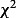	157·71		177·44	
*P*-value	<0·001		<0·001	
Age at first birth (years)				
10–15	0·21	0·79	0·21	0·79
16–20	0·19	0·81	0·24	0·76
21–25	0·15	0·85	0·31	0·69
26–30	0·13	0·87	0·40	0·60
30+	0·12	0·88	0·37	0·63
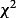	142·22		434·14	
*P*-value	<0·001		<0·001	
Education level				
No education	0·26	0·74	0·16	0·84
Primary	0·21	0·79	0·22	0·78
Secondary	0·16	0·84	0·27	0·73
Higher	0·09	0·91	0·40	0·60
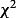	928·75		1350·45	
*P*-value	<0·001		<0·001	
Husband’s/Partner’s education				
No education	0·26	0·74	0·15	0·85
Primary	0·21	0·79	0·21	0·79
Secondary	0·16	0·84	0·27	0·73
Higher	0·10	0·90	0·40	0·60
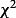	1107·05		1966·13	
*P*-value	<0·001		<0·001	
Occupation				
Unemployed	0·15	0·85	0·22	0·68
White-collar job	0·14	0·86	0·30	0·70
Blue-collar job	0·22	0·78	0·19	0·81
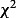	471·63		1121·02	
*P*-value	<0·001		<0·001	
Husband’s/Partner’s occupation				
Unemployed	0·15	0·85	0·30	0·70
White collar job	0·14	0·86	0·33	0·67
Blue collar job	0·21	0·79	0·21	0·79
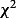	374·13		829·31	
*P*-value	<0·001		<0·001	
Region				
Barisal	0·21	0·79	0·22	0·78
Chittagong	0·17	0·83	0·27	0·73
Dhaka	0·19	0·81	0·26	0·74
Khulna	0·16	0·84	0·26	0·74
Mymensingh	0·18	0·82	0·24	0·76
Rajshahi	0·22	0·78	0·21	0·79
Rangpur	0·20	0·80	0·19	0·81
Sylhet	0·29	0·71	0·17	0·83
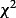	350·54		332·46	
*P*-value	<0·001		<0·001	
Place of residence				
Urban	0·12	0·88	0·36	0·64
Rural	0·22	0·78	0·19	0·81
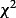	613·26		1762·59	
*P*-value	<0·001		<0·001	
Wealth index				
Poorest	0·31	0·69	0·10	0·90
Poorer	0·24	0·76	0·14	0·86
Middle	0·20	0·80	0·20	0·80
Richer	0·16	0·84	0·27	0·73
Richest	0·07	0·93	0·45	0·55
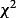	2289·95		4602·31	
*P*-value	<0·001		<0·001	

Table 3 reports the prevalence of women’s underweight and overweight/obesity status across the regions by women’s education level. Overall, we find significant associations (χ^2^ = 378·09, *P* < 0·001; χ^2^ = 189·27, *P* < 0·001) among the variables. Women with higher education have the lowest prevalence of underweight across all the regions except Chittagong and Khulna. Interestingly, the majority of overweight/obese women across all the regions have secondary education. In contrast, we observe the lowest prevalence of overweight/obesity among women with higher education across all the regions other than Barisal, Chittagong and Sylhet. In Barisal, Chittagong and Sylhet, women with no education have the lowest percentage of overweight/obesity.

**Table 3:** Malnutrition among women by education level across regions

	Region	Education level*				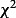	*P*-value
		No education	Primary	Secondary	Higher		
Underweight	Barisal	21·51	43·20	30·67	4·62		
	Chittagong	28·75	29·80	37·15	4·30		
	Dhaka	37·50	32·19	26·54	3·77		
	Khulna	25·63	31·96	36·62	5·79	378·09	<0·001
	Mymensingh	29·82	39·65	24·82	5·71		
	Rajshahi	33·92	29·40	32·48	4·19		
	Rangpur	34·09	31·52	30·16	4·23		
	Sylhet	43·25	33·87	21·06	1·82		
Overweight/obesity	Barisal	7·41	32·78	40·52	19·29		
	Chittagong	12·78	25·04	49·32	12·87		
	Dhaka	17·05	27·44	39·17	16·35		
	Khulna	18·97	27·98	41·14	11·90	189·27	<0·001
	Mymensingh	13·85	33·42	36·80	15·94		
	Rajshahi	19·08	27·79	39·27	13·87		
	Rangpur	20·68	27·54	34·62	17·16		
	Sylhet	18·63	31·93	38·56	10·83		

* All the values represent the percent of malnutrition among women.

Table 4 shows the prevalence of women’s underweight and overweight/obesity status across the regions by their households’ wealth index. Overall, we find significant associations (χ^2^ = 621·17, *P* < 0·001; χ^2^ = 1000, *P* < 0·001) among the variables. Except for Chittagong, we noticed that the proportion of women’s underweight status diminishes with the improvement in the household’s wealth index. Women from either the poorest or poorer households have the highest, while women from the richest households have the lowest prevalence of underweight across all the regions. On the other hand, women from either the richest or richer households have the highest prevalence of overweight/obesity, while women from the poorest households have the lowest prevalence across all the regions other than Sylhet.

**Table 4:** Malnutrition among women by wealth index across regions

	Region	Wealth index*					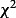	*P*-value
		Poorest	Poorer	Middle	Richer	Richest		
Underweight	Barisal	32·04	32·77	21·69	10·34	3·16		
	Chittagong	18·67	22·97	26·46	22·16	9·74		
	Dhaka	28·48	21·17	18·53	19·76	12·06		
	Khulna	20·73	25·11	25·09	20·04	9·03	621·17	<0·001
	Mymensingh	42·43	31·17	15·20	9·57	1·62		
	Rajshahi	34·55	24·68	21·38	14·18	5·21		
	Rangpur	45·15	27·47	15·16	9·56	2·66		
	Sylhet	33·62	24·37	18·77	13·99	9·25		
Overweight/obesity	Barisal	15·17	16·29	23·08	23·53	18·92		
	Chittagong	5·94	9·78	19·07	25·41	39·80		
	Dhaka	3·22	8·27	11·83	26·09	50·60		
	Khulna	8·27	15·90	19·61	25·45	30·76	1000	<0·001
	Mymensingh	17·75	17·90	19·97	25·63	18·75		
	Rajshahi	11·29	15·11	22·55	25·82	25·23		
	Rangpur	18·95	19·76	25·02	18·82	17·45		
	Sylhet	10·41	9·82	14·43	19·89	45·44		

* All the values represent the percent of malnutrition among women.

Table 1 reports the OR estimates and CI obtained from the multiple logistic regression model of underweight and overweight/obesity on socio-economic and demographic correlates. The Wald - 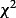 test results reported in Table 1 confirm that our multilevel models for both underweight and overweight/obese indicators are significant as a whole. The likelihood ratio test demonstrates that the multilevel models perform better than the corresponding traditional logistic regression model indicating that the multilevel models significantly improve fit compared with the standard models. Also, the intraclass correlation coefficient values of 0·011 and 0·010 suggest that the models are appropriate for the current study. We find evidence of a significant cluster effect in both our underweight and overweight/obese models, leading to the conclusion that women from different clusters are likely to have a different response on malnutrition indicators.

The risk of being underweight is lower in older age groups compared with younger groups. On the other hand, the odds of women being overweight/obese goes up with the increase in their age. Women aged between 45 and 49 years have a 75 % less chance of being underweight in comparison with women aged between 15 and 19 years. In contrast, the women from the former age group are 8·13 times more likely to be overweight/obese than women from the latter group. Married women have a 45 % lower risk of being underweight and a 59 % higher risk of being overweight/obese compared with single women. The number of total children born apparently has a significant association with women’s underweight and overweight/obesity status. Women who gave birth to three or more children are more likely to be underweight in comparison with women who have no children. Conversely, women with three or more children have a lower risk of being overweight or obese than those with no children. At first birth, women’s age shows a significant relationship with the risk of being underweight and overweight/obese. For example, a woman who gave her first birth at the age between 21 and 25 years has a 26 % higher risk of being underweight and a 20 % lower chance of being overweight/obese than a woman who gave birth at the age of 10–15 years.

On the other hand, both the education levels of women and their husbands/partners have a highly significant association with the underweight and overweight/obesity status of women. The likelihood of women being underweight decreases and overweight/obese increases with women’s increased education level as well as their counterparts. For instance, a woman with higher education is 52 % less likely to be underweight and 48 % more likely to be overweight or obese in comparison with a woman with no education. The chance of being underweight and overweight/obese for a woman with secondary education drops to 63 % less and 54 % more, respectively, compared with a woman with no formal education. Similarly, a woman whose husband or partner receives higher education has a 28 % less chance of being underweight and 41 % more chance of being overweight or obese than a woman with an uneducated husband or partner. Women engaged in blue-collar jobs are 41 % more likely to be underweight but 39 % less likely to be overweight/obese than unemployed women. Women’s white-collar jobs do not show any significant association with their risk of being underweight, but it has a significant association with their overweight/obesity status. Women’s husband’s/partner’s occupation does not appear to have a significant association with their underweight or overweight/obesity status, except the women whose counterparts in blue-collar jobs are 24 % less likely to be overweight than women who have an unemployed husband or partner. Women from Khulna, Mymens and Rangpur have a significantly lower risk, while women from Sylhet have a 71 % higher chance of being underweight than women from Barisal. Women from Rangpur and Sylhet are 10 % and 34 % less likely to be overweight/obese than women from the Barisal region. In comparison, women from the Chittagong division have a 9 % higher chance of being overweight/obese. The place of residence shows a significant association with women’s underweight and overweight/obesity status. Rural women are 1·13 times more likely to be underweight and 23 % less likely to be overweight than urban women. Table 1 also demonstrates that households’ wealth index, regardless of the quintile, significantly influences women’s underweight and overweight/obesity status. The association of the wealth index with the overweight/obesity status is significantly positive, while the relationship is negative on women’s underweight status. Women from the wealthiest households have about three-fourths times less chance of being underweight, while they have a 3·65 times higher risk of being overweight/obese compared with the women from the poorest households. Also, we do not find the multicollinearity issue in our model.

All the negative index values on the left-hand side of the top and bottom panel of Table 5 indicate that underweight status is higher among less-educated women and women from lower quintile households on average. In contrast, the right-hand side of the top and bottom panel shows all positive index values, implying that overweight is more concentrated among higher educated women and women from upper quintile households.

**Table 5:** Education and wealth-related inequalities in women underweight and overweight/obesity status across women’s education and household’s wealth index

Underweight		95 % CI	Overweight/obesity	95 % CI
Education-related inequality				
CI	−0·143***	−0·156, −0·130	0·146***	0·135, 0·157
WI	−0·178***	−0·194, −0·162	0·192***	0·177, 0·207
EI	−0·110***	−0·120, −0·100	0·139***	0·128, 0·150
Wealth-related inequality				
CI	−0·234***	−0·248, −0·220	0·278***	0·266, 0·290
WI	−0·290***	−0·307, −0·273	0·367***	0·351, 0·383
EI	−0·180***	−0·191, −0·169	0·267***	0·256, 0·278

CI , concentration index; WI , wagstaff index; EI , erreygers index.

Significance level:

*** *P* < 0·01.

**Fig. 1:**
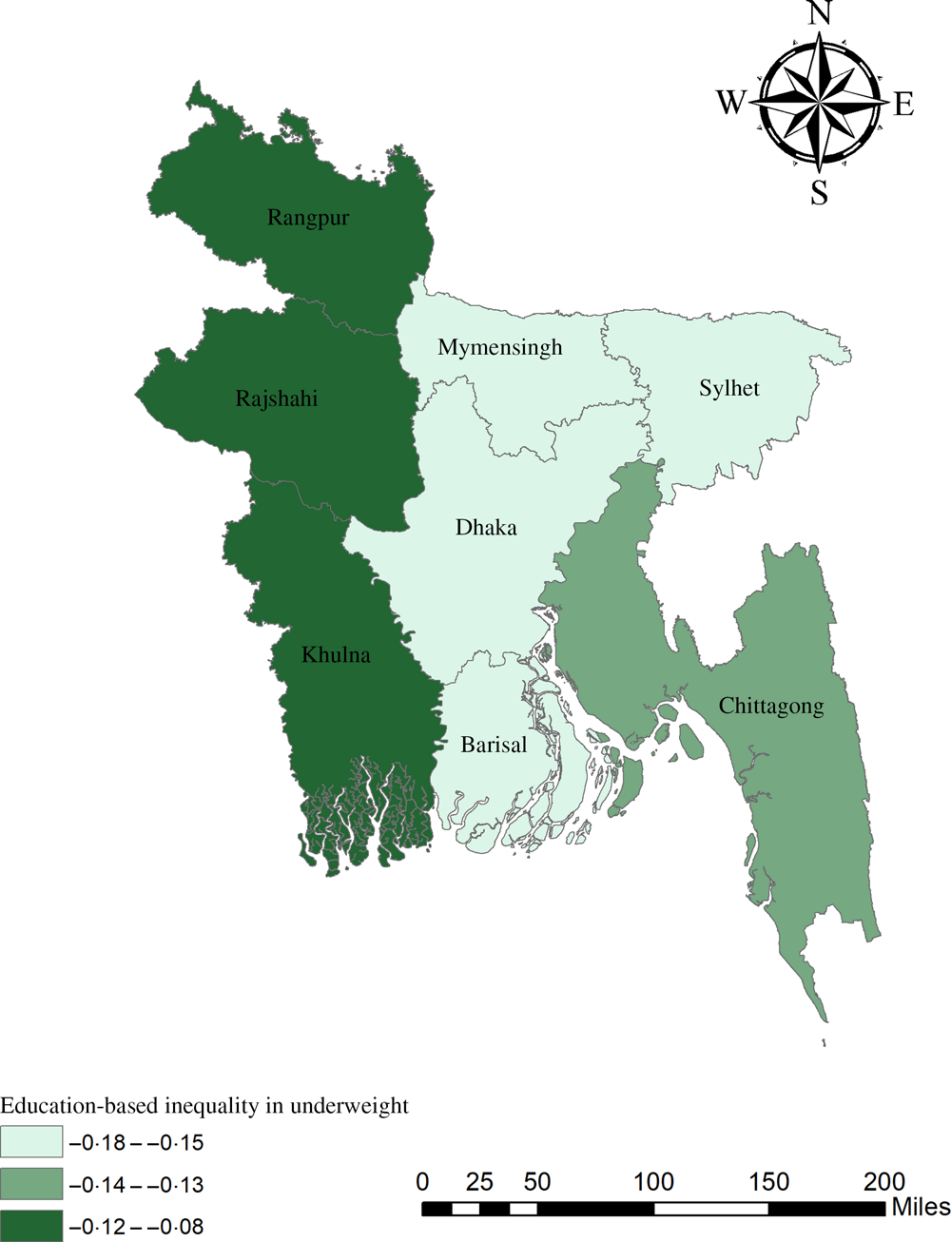
Education-related inequalities in underweight women across the regions of Bangladesh

**Fig. 2:**
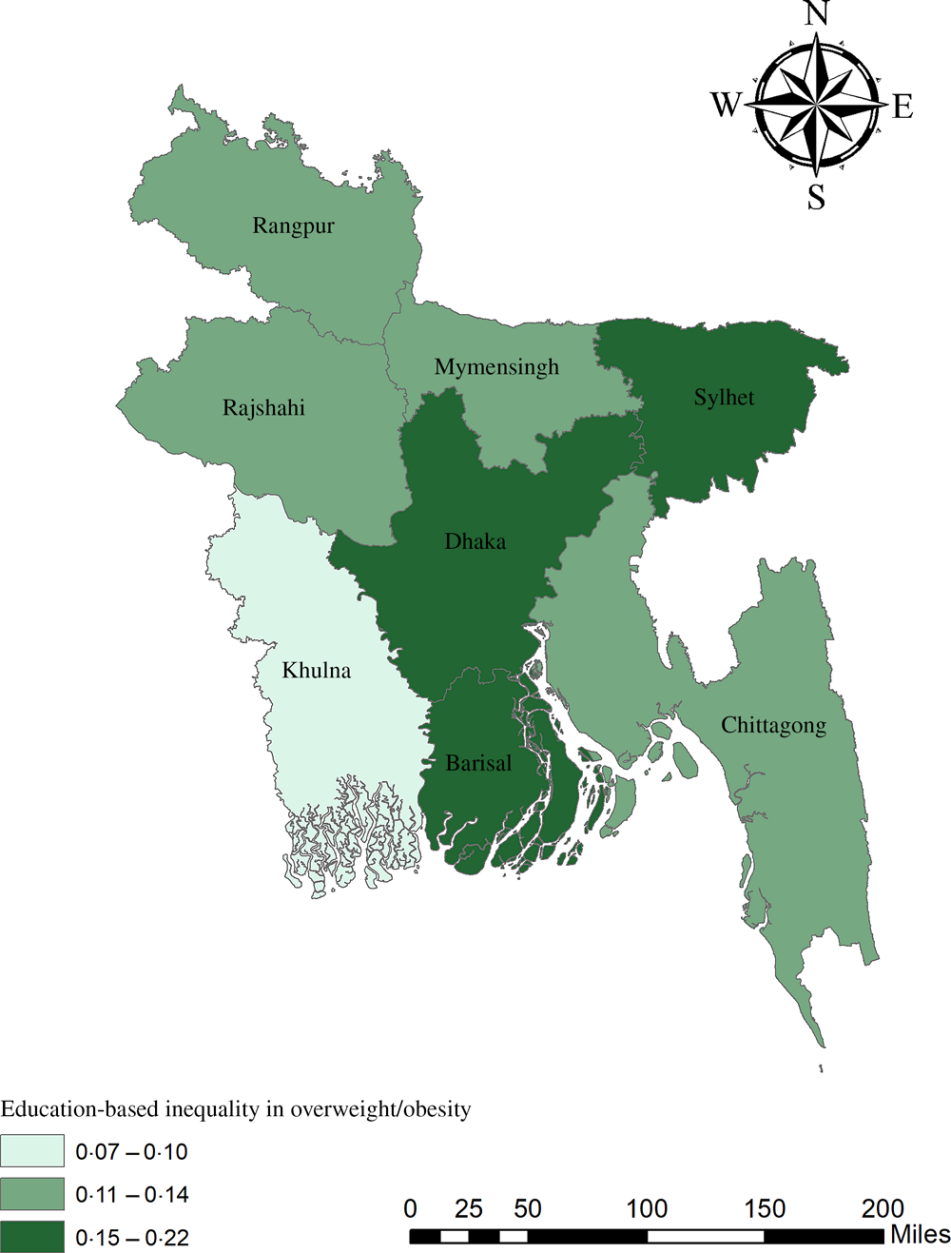
Education-related inequalities in overweight/obese women across the regions of Bangladesh

**Fig. 3:**
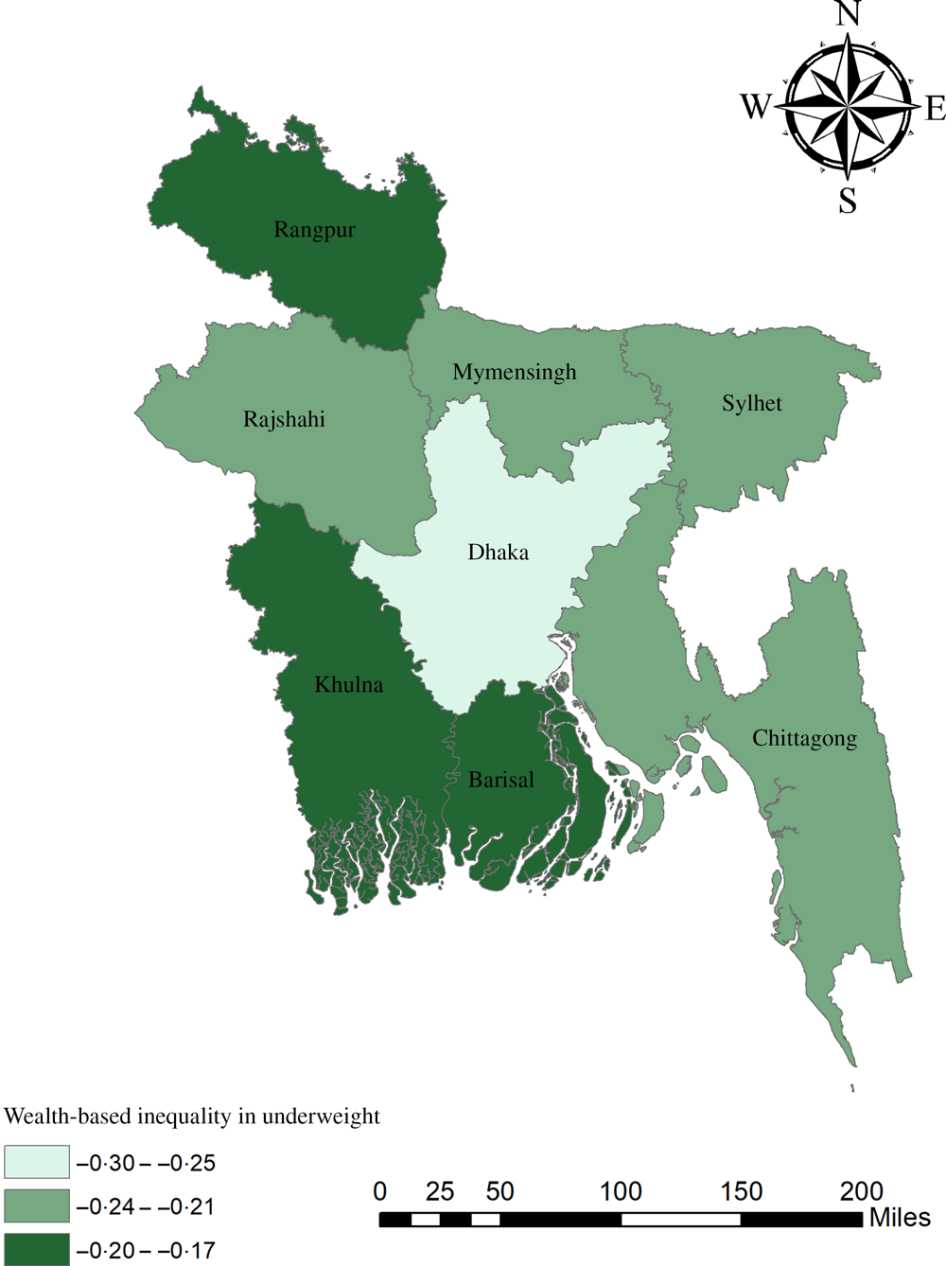
Wealth-related inequalities in underweight women across the regions of Bangladesh

**Fig. 4:**
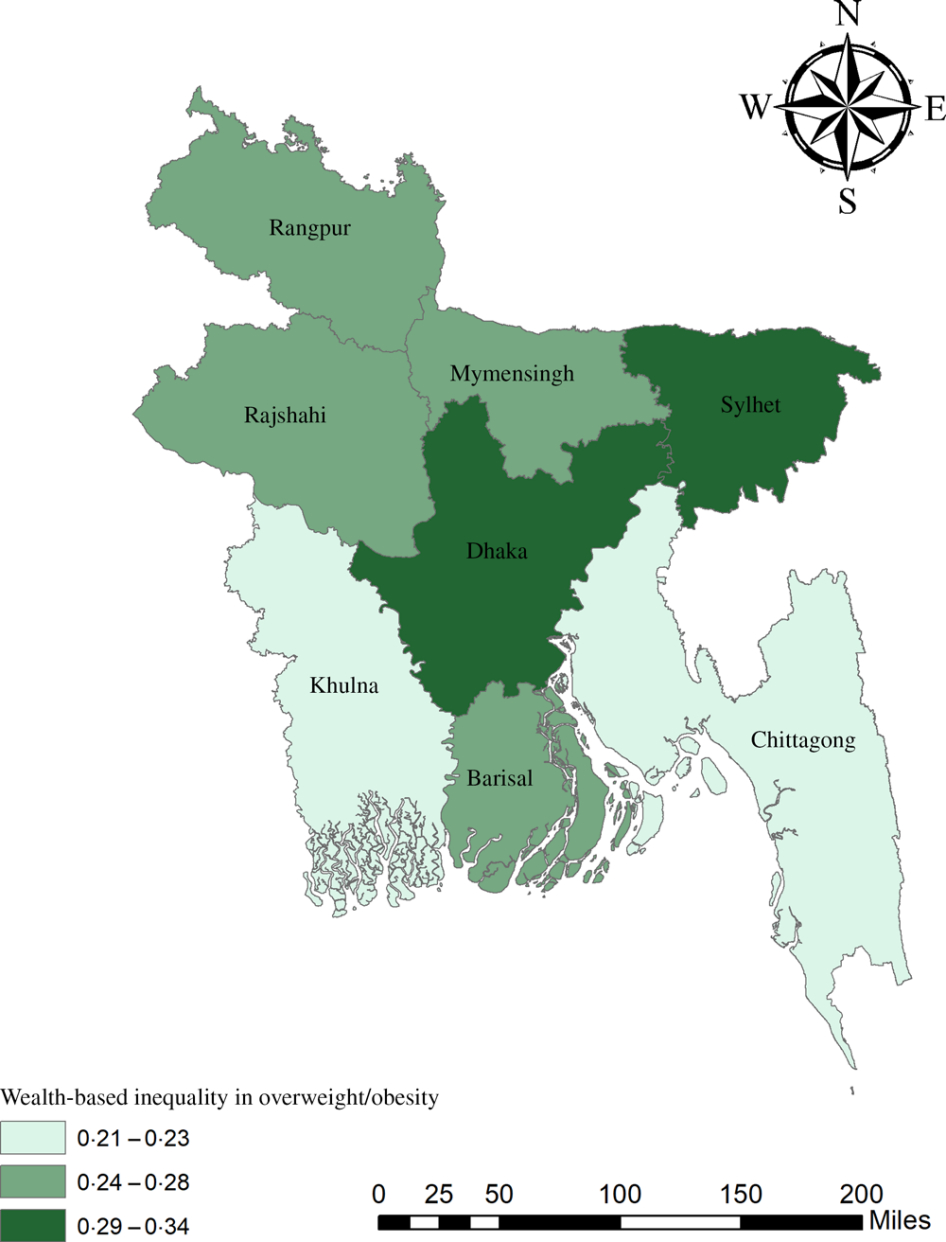
Wealth-related inequalities in overweight/obese women across the regions of Bangladesh

The geographical maps using the concentration index depict the education and wealth-related inequalities in underweight and overweight/obese women across the regions of Bangladesh (Figs. 1–4). Education-related inequality in underweight women is the lowest in the southwestern part and highest in the central areas (Fig. 1). In contrast, education-related inequality in overweight/obese women is the most elevated in the northeastern region, followed by the southern region (Fig. 2). The country’s southwestern part experienced the lowest inequality. Again, the wealth-based inequality in underweight women is the lowest in the southwest part of the country and highest in the central part, followed by the northern region (Fig. 3). On the other hand, wealth-related inequality in overweight/obesity is the highest in the northeastern side, followed by the central area but the lowest in the country’s southwestern part (Fig. 4).

**Fig. 5:**
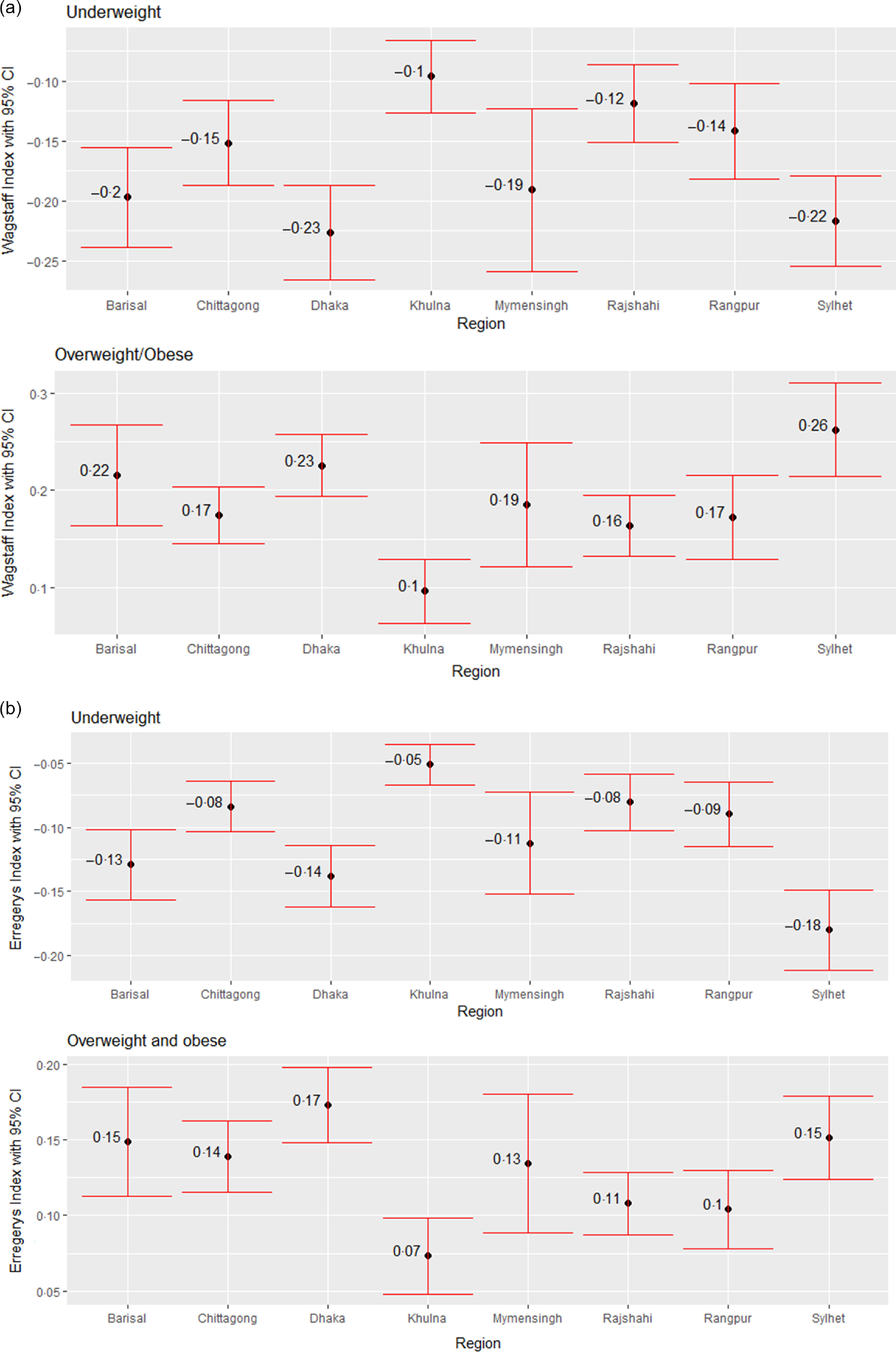
Regional variation in education-related inequalities in underweight and overweight/obese women measured by (a) Wagstaff Index (b) Erregerys Index

**Fig. 6:**
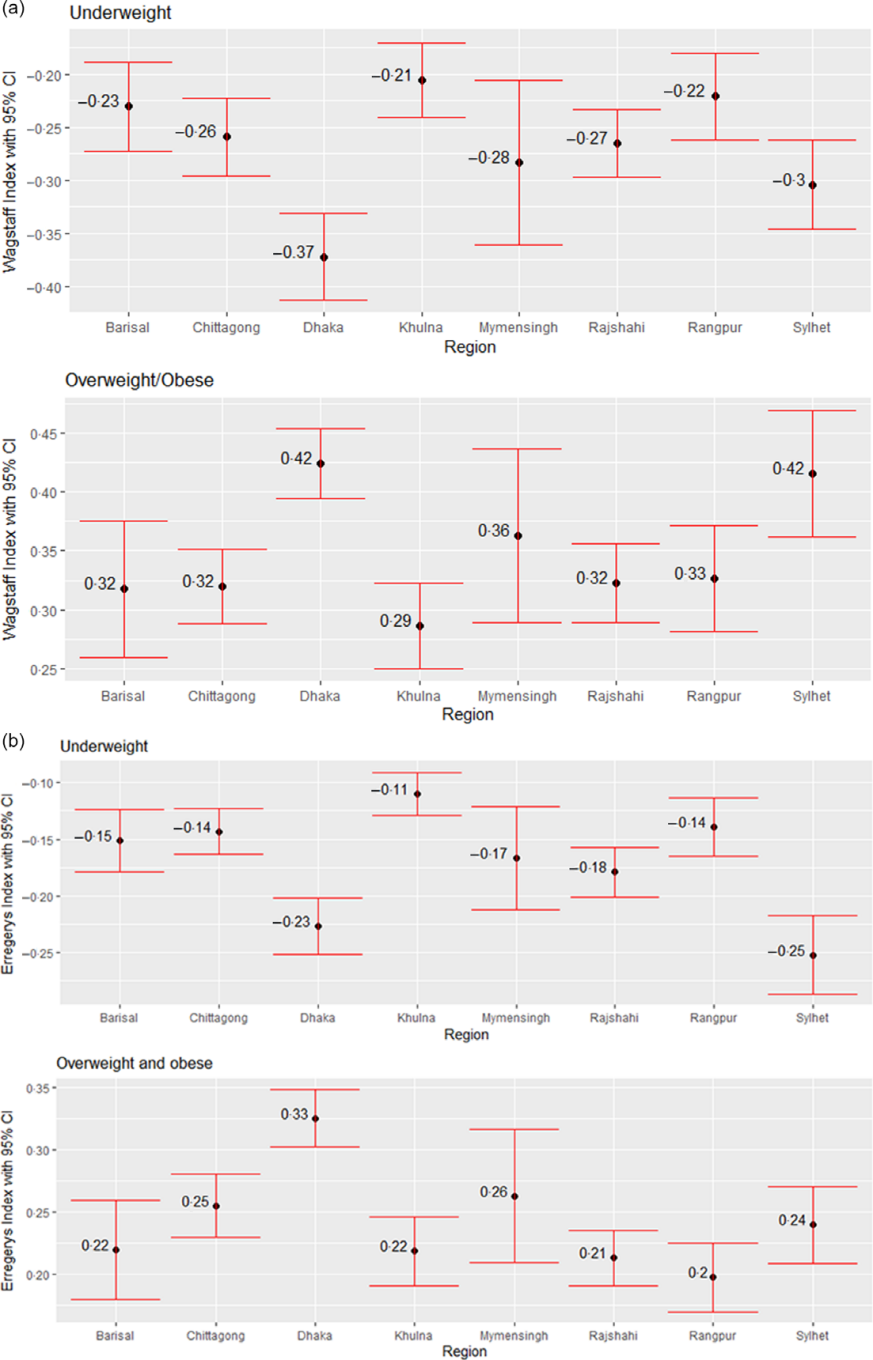
Regional variation in wealth-related inequalities in underweight and overweight/obese women measured by (a) Wagstaff Index (b) Erregerys Index

Next, we assess the education and wealth-related inequality in underweight and overweight/obese women using WI and EI across the eight regions in Bangladesh, as shown in Figs. 5 and 6. The negative values from both indices regarding underweight status suggest that a higher degree of underweight existed among less-educated women and women from poor households. Similarly, the positive values from both indices regarding overweight/obesity status point out that the overweight/obesity problem is more concentrated among the better off in terms of education and wealth status, i.e. higher-educated women and women from wealthy households. Based on both indices, education-related inequality in both underweight and overweight/obese women is the lowest in Khulna (Fig. 5). According to WI, education-related inequality in underweight and overweight/obesity is the highest in Dhaka and Sylhet, respectively, while EI values indicate that women from Sylhet and Dhaka experienced the highest inequality in underweight and overweight/obesity, respectively. Wealth-based inequality in the underweight status of women is the lowest in Khulna in accordance with both WI and EI (Fig. 6). The underweight and overweight/obese women from Dhaka and Sylhet experienced the highest wealth-related inequality based on WI. In contrast, EI suggests that underweight and overweight/obese women from the Sylhet and Dhaka experienced the highest.

**Fig. 7:**
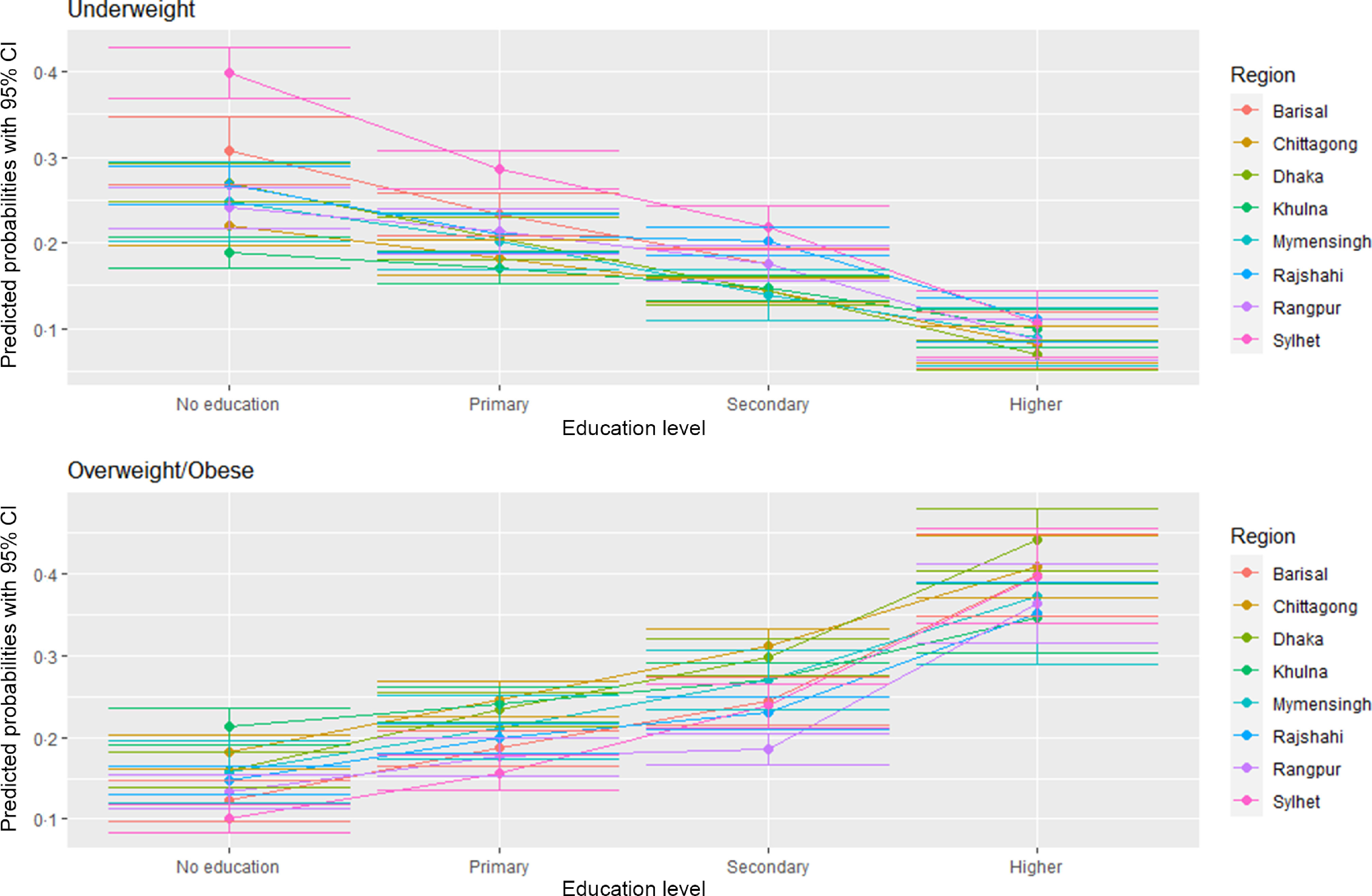
Predicated probabilities from the interactions between education and region

**Fig. 8:**
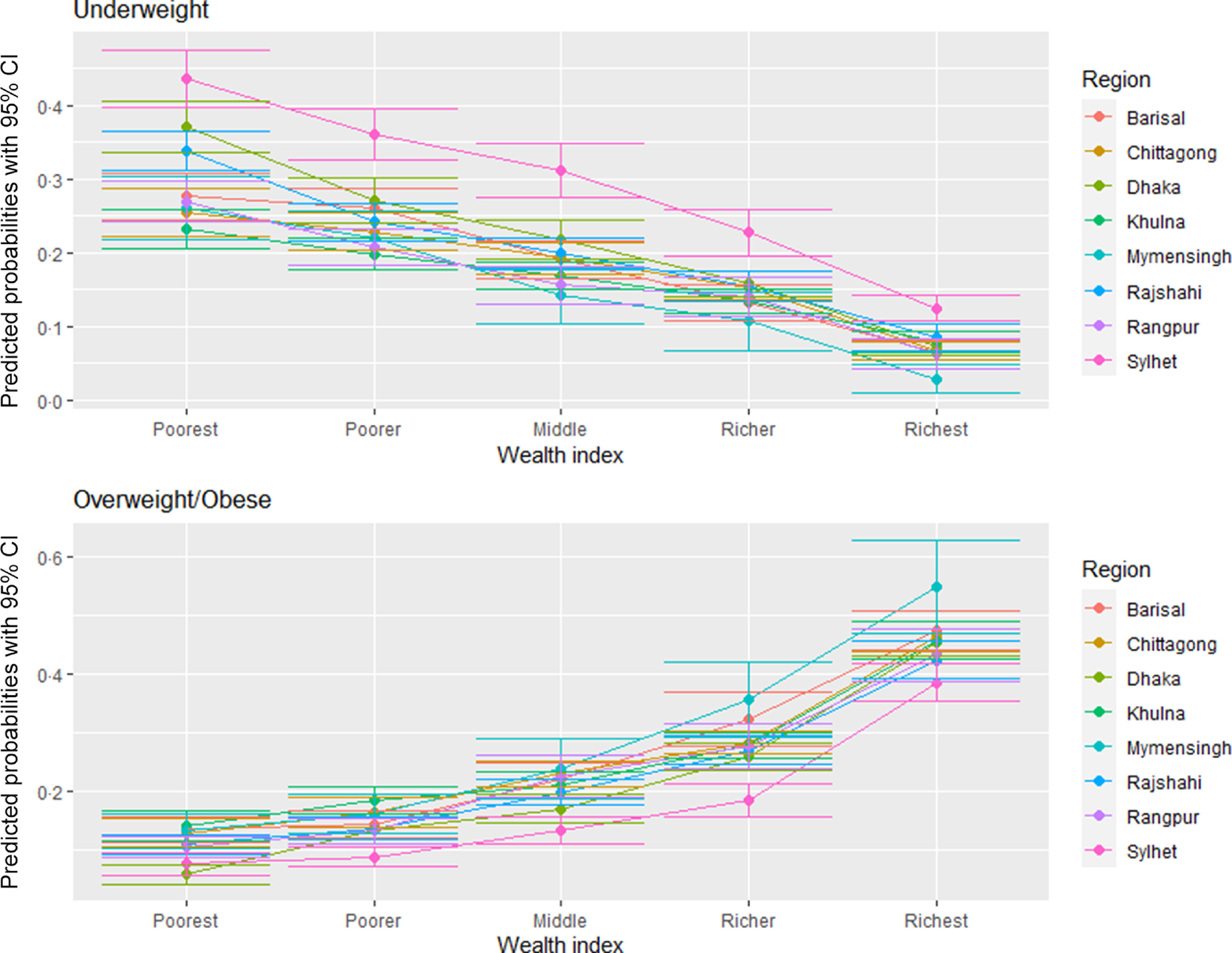
Predicated probabilities from the interactions between wealth and region

Figures 7 and 8 delineate the predicted probabilities of underweight and overweight/obesity by education and wealth index across the regions. The predicted probability of being overweight/obese is the highest among the richest and higher educated women than the poorest and ones with no formal education (Fig. 7). The predicted probability of being overweight is the highest among women from Dhaka across all education levels except no education. In contrast, women from Sylhet have the highest likelihood of being underweight across all education levels. Figure 8 demonstrates that the likelihood of being an underweight woman is higher in the poorest households relative to the wealthiest ones across the regions. Simultaneously, the predicted probability of being overweight/obese goes up with the improvement in the wealth index across the regions. Again, women residing in Sylhet have the highest predicted chance of being underweight across all the wealth index quintiles.

## Discussions

The current study finds that a set of predictors, such as women’s age, current marital status, total children ever born, age at first birth, education level, husband’s/partner’s education level, blue-collar job, place of residence, regions and wealth index, have a significant strong association with underweight and overweight/obesity status of reproductive women.

We observe increased odds of being underweight among younger women while the prevalence and odds of being overweight/obese augmented as they got older. This implies women’s age has a negative association with their underweight status but positive with overweight/obesity. Some previous studies conducted in Bangladesh and other countries demonstrated these relationships^(37–40)^. The possible explanation can be that ageing is a key determinant of many non-communicable diseases, including obesity^(41)^. It has a significant connection with substantial body composition changes since fat mass escalates after age 30 while fat-free mass gradually depresses^(42,43)^. Also, as people get older, they feel more reluctant to do physical activity while maintaining the same or increased intake of nutritious food. Women who are married during the survey tended to be underweight less and overweight more. One of the reasons can be married women usually care less about their weight gain. Also, sometimes they use different hormonal contraceptives, e.g. implant and intrauterine devices (IUD), to control birth, potentially increasing their weight, according to past studies^(44,45)^. The number of total children born is positively associated with women’s underweight status while negatively with overweight/obesity. Two past studies advocated these results^(12,20)^. These findings suggest that giving birth to many children can pose a long-term threat to the mother’s health and leave them malnourished. Therefore, a woman needs more micronutrients and sufficient protein-energy intake after becoming a mother to keep her fit and support her child’s development. Otherwise, low energy and slower brain development may hamper the productive capacity of both mother and children. Our results suggest that the more women gave their first birth late, the less they could be underweight while having a higher risk of being overweight/obese. This association may have a potential linkage with women’s age.

Our findings imply that women and their husband’s/partner’s education levels have a significant association with women’s double burden of malnutrition. The likelihood of women being underweight dampens and overweight/obese goes up with their increased education level as well as their counterparts. Previous studies from the context of Bangladesh and other developing countries demonstrate that higher educated women have a higher risk of being overweight or obese^(9,38,46)^. Occupational sitting time is higher in persons who have higher education and prefer desk jobs, resulting in an increased risk of overweight and obesity for them^(47)^. Another study reports that education level has a positive association with leisure time and a negative relation with the work index of habituated physical activity^(48)^. Women engaged in blue-collar jobs are less likely to be overweight than those who are unemployed or involved in white-collar jobs. This again pointed to the higher odds of being overweight/obese among women who are less likely to be engaged in physically demanding work. The current study also reports that rural women are more likely to be underweight, where the proportion of overweight/obese women is almost double in urban areas compared with rural. Our findings are in line with a couple of previous studies^(38,49)^. With rapid economic growth and growing urbanisation, consumers’ preferences have shifted to processed and fast foods that are nutrient-poor, energy dense and high in fat and sugar, paving the way to overweight and obesity. Similarly, lack of access to nutritious intake and income deficit propels rural women to undernutrition. Our findings corroborated that woman are less likely to be underweight and more likely to be overweight/obese with the amelioration in their household’s position in the wealth index. This outcome is consistent with a number of past studies conducted in developing countries^(37,38,50,51)^. Our analysis finds evidence of significant differences in the women’s underweight and overweight/obesity status across regions. We find that women in the northwestern and northeastern regions, such as Rajshahi, Rangpur and Sylhet, are more likely to be underweight. Previous regional studies in Bangladesh report similar outcomes regarding women’s underweight status^(13)^. In contrast, the overweight/obesity status is significantly higher in the central, southeastern and southwestern parts of the country, i.e. Dhaka, Chittagong and Khulna, which is consistent with the findings of a previous study^(52,53)^.

We find significant associations between women’s double burden of malnutrition, their education level and their household wealth index. The important contribution of this paper lies in investigating education and wealth-related inequalities in underweight and overweight/obesity among reproductive women across the whole country and its regions. Our findings suggest that underweight status is higher among less-educated women and women from poor households, whereas overweight is more concentrated among higher educated women and women from rich households. Our analysis suggests that the southwestern region of the country, i.e. Khulna shows minimum education and wealth-related inequalities in women’s double burden of malnutrition. This result is robust across all three indices used in this paper. Health and nutritional surveillance project for development demonstrated that malnutrition in children and mothers is lower in Khulna^(54)^. The central region, i.e. Dhaka and the northeastern part of the country, i.e. Sylhet, apparently experienced the highest education and wealth-related inequalities in underweight and overweight/obesity among women of reproductive age. This finding is consistent with one of the past studies that finds pro-wealthy inequalities in antenatal services and delivery care services are the highest in Sylhet and Dhaka and the lowest in the Khulna and Rangpur^(29)^. Dhaka is one of the most densely industrial regions of the world with social and economic diversity. Most of the slum areas are also located in this geographical region, which resulted in the highest education and wealth-related inequalities in malnutrition among women^(55)^. Urbanisations create various restaurants, food parks and supermarkets, which gained popularity and changed people’s diet habits^(56)^. On the other hand, individuals struggle to manage proper meals a day in most of the slum areas in Dhaka city, resulting in undernutrition among adult individuals^(57)^. Therefore, education and wealth-related inequalities are the highest in Dhaka. Several past studies conducted in Bangladesh find that income and education-related disparities in individual’s underweight and overweight prevalence increase in Sylhet, which is in line with our findings^(58,59)^. Traffic congestion, transportation problem, geographic remoteness, limited access to health care, education, food and jobs could be the reason for inequalities in particular regions^(60–63)^.

We observe that women’s education level has a negative relationship with underweight status and a positive relationship with overweight/obesity across all the regions. Similarly, the wealth index is negatively associated with underweight and positively with overweight/obesity across all the regions. The differences in predicted probabilities of being underweight between regions shrank at higher education levels and richest quintile. The differences in the likelihood of being overweight/obese diminish at the primary education level and lower quintile of households. The predicted probability of being underweight is the highest among women from Sylhet across all the education levels and wealth index quintiles.

### Limitations of the study

The current study has several limitations that warrant consideration in future studies in the context of Bangladesh. First, the current study follows a cross-sectional design that does not make causal inferences between women’s malnutrition outcomes and socio-economic status. Second, BDHS does not collect data on some essential information, e.g. food intake, physical condition and sitting time, which prevents us from controlling them in our model. Third, we restricted our sample to the ever-married women aged 15–49 years. Hence, the current study might not reflect the complete nutritional status of women. Also, we do not control for regional factors, e.g. temperature, precipitation, population and health care facilities in our model.

## Conclusions

This paper finds that women’s age, current marital status, total children ever born, education level, husband’s/partner’s education level, place of residence and wealth index are significantly associated with women’s underweight and overweight/obesity status. Also, we find evidence of a significant cluster effect, indicating community variation in the data. To the best of our knowledge, as of today, this is the first paper to investigate the regional variation of education and wealth-related inequalities in malnutrition among women in Bangladesh. We find that underweight status is higher among less-educated women and women from poor households, whereas overweight/obesity is more concentrated among higher educated women and women from wealthy households. The southwestern region of the country demonstrates lower education and wealth-related inequalities in malnutrition among women. In contrast, the central and the northeastern areas apparently experience the highest education and wealth-related inequalities in malnutrition among women. The regional differences in predicted probabilities of being underweight are lower at higher education levels and the wealthiest quintile. In contrast, the differences in overweight/obese diminish at the primary education level and lower quintile households. Malnutrition has a more notable impact on women than men as it may affect their own health as well as their children’s health. Our findings strengthen the evidence base for effective regional policy interventions to mitigate education and wealth-related inequalities in malnutrition among women across regions. There is a need to develop regional awareness programmes to advertise the importance of sufficient nutrition and a balanced diet with a view to reaching the full potential of their development and economic success. Also, we need to establish regional monitoring cells, especially in the underprivileged areas, to ensure proper health and nutrition facilities in those regions. The government and local authorities should take initiatives to resolve traffic congestion and transportation problems and increase health care, education, food and job facilities in underprivileged regions to mitigate inequalities. The current study would yield thoughtful insights to local and international entities, government, policymakers and researchers into the problem that policymakers may take into account while formulating strategies to reduce regional disparities in malnutrition among women. However, further research is recommended to examine the potential sources of regional variation in the prevalence of women’s malnutrition in the country.
